# UV-Accelerated Photocatalytic Degradation of Pesticide over Magnetite and Cobalt Ferrite Decorated Graphene Oxide Composite

**DOI:** 10.3390/plants10010006

**Published:** 2020-12-23

**Authors:** Asma Tabasum, Mousa Alghuthaymi, Umair Yaqub Qazi, Imran Shahid, Qamar Abbas, Rahat Javaid, Nimra Nadeem, Muhammad Zahid

**Affiliations:** 1 Department of Chemistry, University of Agriculture Faisalabad, Faisalabad 38040, Pakistan; bintetabasum1112@gmail.com (A.T.); nimranadeem692@gmail.com (N.N.); 2 Biology Department, Science and Humanities College, Shaqra University, Alquwayiyah 11726, Saudi Arabia; malghuthaymi@su.edu.sa; 3 Department of Chemistry, College of ScienceUniversity of Hafr Al Batin, P.O Box 1803, Hafr Al Batin 39524, Saudi Arabia; umairqazi@uhb.edu.sa; 4 Environmental Science Centre, Qatar University, Doha P.O. Box 2713, Qatar; ishahid@qu.edu.qa; 5 Institute for Chemistry and Technology of Materials, Graz University of Technology, Stremayrgasse 9, 8010 Graz, Austria; qamar.abbas@tugraz.at; 6 Fukushima Renewable Energy Institute, National Institute of Advanced Industrial Science and Technology, AIST, 2-2-9 Machiikedai, Koriyama, Fukushima 963-0298, Japan; rahat.javaid@aist.go.jp

**Keywords:** acetamprid, wastewater treatment, response surface methodology, magnetic ferrites

## Abstract

Pesticides are one of the main organic pollutants as they are highly toxic and extensively used worldwide. The reclamation of wastewater containing pesticides is of utmost importance. For this purpose, GO-doped metal ferrites (GO-Fe_3_O_4_ and GO-CoFe_2_O_4_) were prepared and characterized using scanning electron microscopy, X-ray diffraction and Fourier transform infrared spectroscopic techniques. Photocatalytic potentials of catalysts were investigated against acetamiprid’s degradation. A detailed review of the parametric study revealed that efficiency of overall Fenton’s process relies on the combined effects of contributing factors, i.e., pH, initial oxidant concentration, catalyst dose, contact time, and acetamiprid load. ~97 and ~90% degradation of the acetamiprid was achieved by GO-CoFe_2_O_4_ and GO-Fe_3_O_4_, respectively during the first hour under UV radiations at optimized reaction conditions. At optimized conditions (i.e., pH:3, [H_2_O_2_]: 14.5 mM (for Fe_3_O_4_, GO-Fe_3_O_4,_ and GO-CoFe_2_O_4_) and 21.75 mM (for CoFe_2_O_4_), catalysts: 100 mgL^−1^, time: 60min) the catalysts exhibited excellent performance, with high degradation rate, magnetic power, easy recovery at the end, and efficient reusability (up to 5 cycles without any considerable loss in catalytic activity). A high magnetic character offers its easy separation from aqueous systems using an external magnet. Moreover, the combined effects of experimental variables were assessed simultaneously and justified using response surface methodology (RSM).

## 1. Introduction

Pesticides have been widely used worldwide to meet the food demands, but these exhibit persistency, bioaccumulation, and easy transportation over long distances from their sources, thus posing a serious threat to environmental habitats [1]. Neonicotinoid is a fast-growing class of commercialized pesticides. They are derived from nicotine and are used as alternatives to synthetic, extremely hazardous pesticides [2]. The extensive use of these pesticides would cause serious threats to the terrestrial and aquatic environment [3]. These are water-soluble, photo-stable, and persistent to biological degradation. The active compounds and major by-products enable their transference in surface water as well as underground water. The extensive use of pesticides in the agriculture sector not only contaminates the crops but also affects the environment on the whole [4]. A third-generation insecticide of this group is acetamiprid. It is widely used in a variety of crops such as fruit trees, tobacco, melons, leafy vegetables, wheat, and cotton to control sucking insects like aphids, moths, beetles, etc. It is soluble in most organic solvents and shows increased solubility in water (i.e., 25 g L^−1^), and this high-water solubility increases its presence in surface water [[5,6,7,8]]. The systematic name of acetamiprid is (E)-N1-[(6-chloro-3-pyridyl) methyl]-N 2- cyano- N1-methylacetamidine, CAS No: 135410-20-7. Since its (acetamiprid’s) arrival in the global market in early 1990s, it has been registered in more than 125 countries throughout the world. Its excellent performance has surged its usage accounting for 30% of global insecticide market [9]. Due to high water solubility, more than 80% residues of acetamiprid persisted in the soil of treated crops, ultimately entering into surface water or groundwater. Recently, a survey of nine countries pointed out that more than 80% surface waters have been contaminated with it up to the level of 0.14 µg/L to 18 µg/L. This concentration level has been proved to be sublethal to aquatic plants and animals, especially to aquatic arthropods [10].

Despite the extended use of acetamiprid, its degradation has been less frequently investigated. Earlier, studies revealed that persistent organic pollutants have been treated with many conventional processes such as chemical, physical, and biophysical processes but proved inefficient. In recent years, effective and viable treatment technologies widely used were Advanced Oxidation Processes (AOPs) [11,12]. These Processes involve the in situ production of oxidizing species such as hydroxyl radicals (**^·^**OH). Versatile mechanisms under AOPs are involved in the production of these reactive species such as photocatalysis. Oxides of Zinc, Titanium, Iron, etc. are widely used as photocatalysts, but their wide bandgap limits their application, as they absorb high energy photons near the UV region [13,14]. To promote efficient utilization of sunlight, the extensive attraction has been diverted towards the development of photocatalysts with narrow bandgap energy. One of the simple AOPs is the Fenton process, which employs Fe (II) ions and H_2_O_2_ to produce ·OH radicals, which are highly reactive and nonselective species, reflecting high potential to mineralize hazardous and biorefractory organic pollutants present in water bodies.

The efficiency of the homogenous Fenton process was enhanced by avoiding sludge formation and promoting cyclization of Fe^3+^ to Fe^2+^ [15]. The issue regarding the separation of dissolved iron was solved by introducing a heterogeneous Fenton process [16]. It utilizes the heterogeneous iron catalysts, the unique advantages of which are their easy separation from treated water, thus avoiding sludge formation [17,18]. In this connection, the usage of iron oxide (Fe_3_O_4_) in its proper chemical assembly and valence state received much attention owing to its unique properties such as magnetic behavior, high surface to volume ratio, superior resistance towards corrosion, low toxicity, and high environmental stability [19]. On the other hand, several factors restrict its application on large scale such as its agglomeration in solution, particle size, and spatial arrangement of constituent species in the crystal structure [20]. Hydrophobic forces on the catalyst’s surface are responsible for the agglomeration of these particles rendering a remarkable decline in catalytic efficiency which consequently increases cost and time consumption in the separation step [21]. This problem was resolved by impregnating the catalyst with suitable carbonaceous support. The type and nature of support play a vital role. Graphene oxide is one of the ideal candidates due to its versatile catalytic and adsorptive properties [22,23,24].

In the present study, iron ferrite and cobalt ferrite were prepared by co-precipitation and hydrothermal methods, respectively, while Hummer’s method was adopted for graphene oxide synthesis. Due to exceptionally promising applications and unique properties [25], the cobalt ferrite has been used for photocatalytic degradation of Acetamiprid pesticide. The degradation potential of iron ferrite, cobalt ferrite, and their graphene oxide composites was compared to check the best responsive photocatalyst among them. Characterization of ferrites and their composites was carried out using Scanning Electron Microscopy (SEM), X-ray Diffraction technique (XRD), and Fourier Transform Infrared spectroscopy (FTIR). The effects of certain experimental factors such as initial dose of pesticide, pH, catalyst dose, oxidant concentration, and UV-light exposure time were studied to enhance the process’s efficiency. Ferrites and their GO composites were magnetically separable, and their reusability was studied for the degradation of pollutants cyclically. Further, three kinetic models were chosen for degradation study including the 1st order kinetic model, 2nd order kinetic model, and BMG kinetic model. The central composite design for response surface methodology was used as a statistical tool to understand the mutual effects of influencing parameters like catalysts dose, oxidant dose, and pesticide concentration.

This study aims to present useful scientific information on the effectiveness of magnetic composite based AOPs for the remediation of recalcitrant organic compounds especially neonicotinoid pesticide.

## 2. Results

### Characterization

Characterization (SEM, XRD, and FTIR) of magnetite and its GO-based catalyst, sonochemical synthesized, was precisely reported earlier [5]. As explained before, moderate ultrasonic irradiation involved in co-precipitation reaction, yielded smaller sized, magnetically improved Fe_3_O_4_ particles with narrow size distribution.

**Figure 1:**
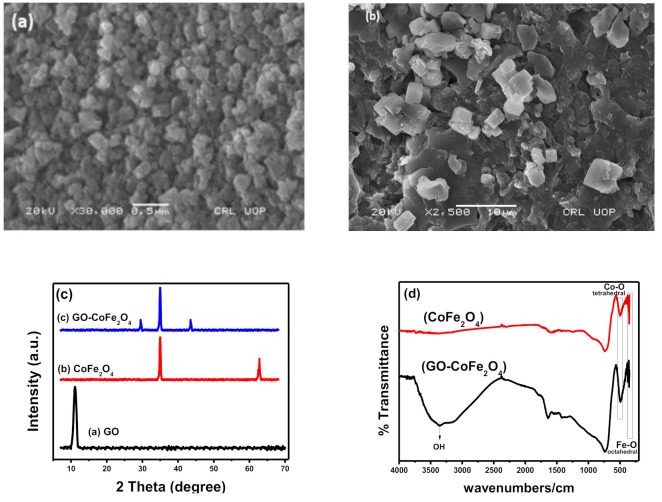
Characterization analysis of catalysts; SEM images of (**a**) CoFe_2_O_4_, (**b**) GO-CoFe_2_O_4_, (**c**) XRD patterns, and (**d**) FTIR spectra CoFe_2_O_4_ alone and its GO-based composite.

Figure 1a,b shows the SEM images of CoFe_2_O_4_ and GO-CoFe_2_O_4_ respectively, exhibiting the comparative morphologies of both (CoFe_2_O_4_ and its composite). It is quite clear from the SEM images that the composite is formed due to the firm linkage of embedded ferrite cubical particles on the highly exposed surface of exfoliated graphene oxide.

Successful composite synthesis is also supported by XRD peaks (Figure 1c) and FTIR spectra (Figure 1d). In the XRD pattern of CoFe_2_O_4_, the characteristic peaks at 35.64° and 62.77° corresponds to the (311) and (440) crystal planes of copper ferrite, whereas in GO-CoFe_2_O_4_, a broad shift of plane can be observed. The disappearance of GO peak in GO-CoFe_2_O_4_ may be due to the reduction of rGO, or it may be due to the destruction of regular stacking of GO sheets by the crystal growth of CoFe_2_O_4_ between the layers [26,27].

FTIR also confirmed the developed metal-oxygen bonds during the formation of ferrites. Two absorption bands appearing below 600 cm^−1^ are characteristic features of ferrites. The bands at higher and lower frequencies are assigned to stretching vibrations of tetrahedral metal ion-oxygen ion (M_tet_-O) and octahedral metal ion-oxygen ion (M_Oct_-O), respectively. While in composite, the band near 3400 cm^−1^ endorses the stretching vibration of the hydroxyl group present on the GO surface.

**Figure 2:**
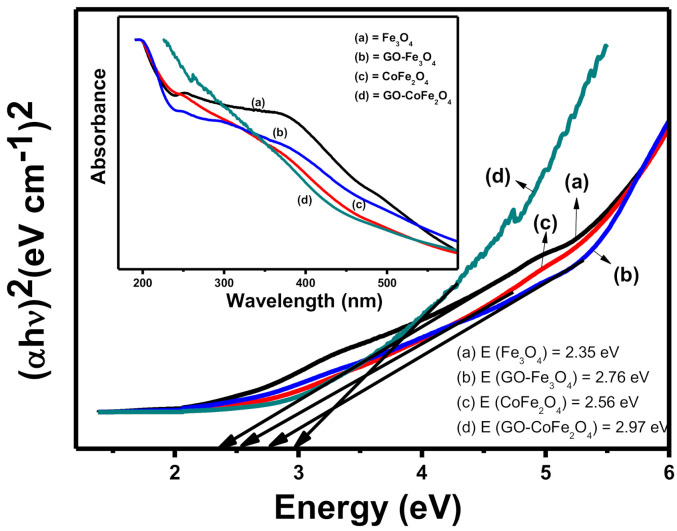
Bandgap energy estimation of catalysts using Tauc plot method (inset UV-Visible absorption spectra).

UV-Visible spectroscopy was carried out to check the bandgap energies of catalysts using Tauc plot method. The results obtained are presented in Figure 2. The band gap energies (in eV) of Fe_3_O_4_, GO-Fe_3_O_4_, CoFe_2_O_4_, and GO- CoFe_2_O_4_ were found to be 2.35, 2.76, 2.56, and 2.97, respectively. The increase in band gap energies from metal ferrites to their graphene oxide composites was observed [28,29]. This ensures the better charge (electron hole pair) separation in the composites and results in improved photocatalysis as compared to the pristine metal ferrites.

## 3. Discussion

### 3.1. Degradation Potential of Catalysts against Acetamiprid’s Remediation

#### 3.1.1. Effect of pH

**Figure 3:**
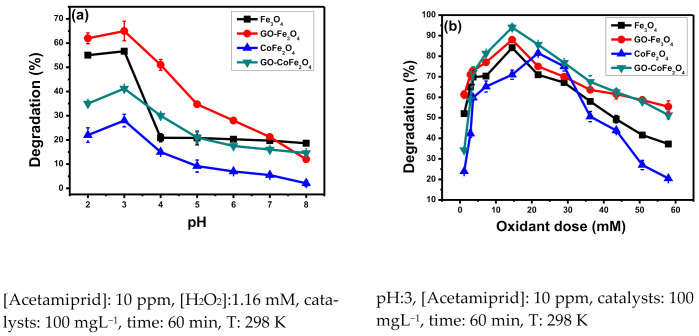

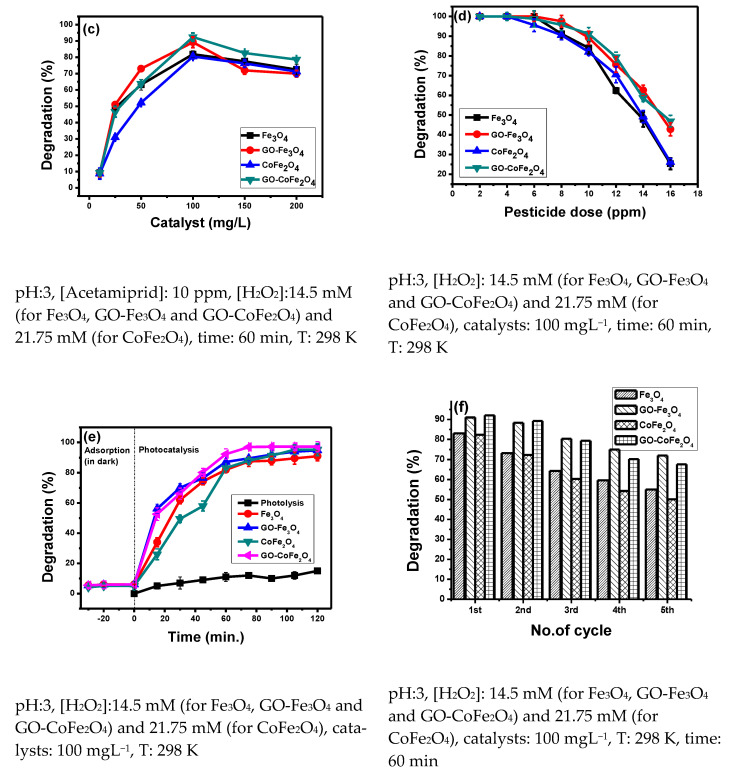
Optimization of (**a**) pH, (**b**) oxidant concentration, (**c**) catalyst dose, (**d**) pesticide load, (**e**) time of UV irradiation, and (**f**) reusability potential up to 5 consecutive runs against degradation of acetamiprid using Fe_3_O_4_, GO-Fe_3_O_4_, CoFe_2_O_4_, and GO-COFe_2_O_4_.

Considering pH as one of the influential parameters in catalysis, 7 Acetamiprid solutions (10ppm each) with pH values determined initially (2 to 8) were selected. The pH of different solutions was maintained using 0.1 M hydrochloric acid and 0.1 M sodium hydroxide solutions. In Figure 3a, the obtained results in terms of % degradation as a function of pH have been presented. It can be noticed that all the catalysts (Fe_3_O_4_, GO-Fe_3_O_4_, CoFe_2_O_4_, and GO-CoFe_2_O_4_) put their maximum degradation potential at 3 pH (highly acidic condition). The catalyst’s degradation potentials (in all cases) have been dropped when approaching alkaline pH from pH 3 (e.g., in case of Fe_3_O_4_ 57% (at pH 3) to 21% (at pH ≥ 4), GO-Fe_3_O_4_ 65% (at pH 3) to 18% (at pH 8), CoFe_2_O_4_ 40% (at pH 3) to 18% (at pH 6), and GO-CoFe_2_O_4_ 27% (at pH 3) to 5% (at pH ≥ 7)). At higher pH (more than 7), a gradual decrease in catalytic activity is due to the unwanted reaction of ferrous ions and excess hydroxyl ions (present in the solution (Equation (1)). This reaction will lead to the formation of yellowish-brown ferric hydroxide sludge, which ultimately will cover the active catalyst’ sites and hence contribute to the reduction of overall efficiency. This is the situation when surface-bound-transportation of molecules and other charge carriers is hindered.

(1) $$2HO^{-}+Fe_{surf}^{2+}\to Fe{\left(OH\right)}_{2surf}$$

Moreover, hydrogen peroxide molecules, at alkaline pH, would more likely be auto decomposed (Equations (2) and (3)). Therefore, no more active reagents (such as catalyst and hydroxyl radicals) would be available to conduct the degradation reaction effectively [5].

(2) $$HO^{-}+H_{2}O_{2}\to HO_{2}^{-}+H_{2}O$$

(3) $$\;H_{2}O_{2}+HO_{2}^{-}\to O_{2}+H_{2}O+HO^{-}$$

when hydrogen ion concentration is very high in the pesticide solution, at acidic conditions (pH closer to 3), more hydroxyl radicals are susceptible to be attacked upon by H^+^ ions present in the solution, forming a relatively stable specie H_3_O_2_^+^. Consequently here, during Fenton’s reaction, the catalyst’s performance is retarded, because hydroxyl radicals become limiting reactants. It is evident from Figure 3a that acetamiprid’s degradation is highest at pH 3 so, it is taken as optimized pH (in all cases).

(4) $$H_{2}O_{2}+H_{2}O_{2}\to 2H_{2}O+\;O_{2}$$

#### 3.1.2. Effect of Oxidant Dose

In the heterogeneous photo-Fenton process, the role of oxidant (H_2_O_2_) is also very decisive because it acts as an accelerator for the photocatalysts. The effect of oxidant was investigated using hydrogen peroxide (H_2_O_2_) dose-ranging from 1.16 to 58 mM. The results are represented in Figure 3b. Here before the discussion, two opposing factors must be considered, i.e.,

(a) If oxidant in the photochemical reaction is added in a sufficient amount, it will absorb photons from the environment and help in promoting the rate of photolysis reaction occurring at the catalyst’s surface (Equation (5)). Consequently, more hydroxyl radicals would be available for pollutant degradation. Hydrogen peroxide not only enhances the Fenton’s reaction but also promotes the reusability of the catalyst (Equation (6)).
  (5) $$H_{2}O_{2}+Fe^{3+}+hv\to Fe^{2+}+HO_{2}^{■}+H^{+}$$
  (6) $$H_{2}O_{2}+Fe^{2+}\to Fe^{3+}+HO^{■}+HO^{-}$$
(b) On the other hand, hydroxyl radicals can efficiently react with hydrogen peroxide, so the excess of hydrogen peroxide will act as a hydroxyl radical scavenger (Equation (7)), which will lead to a decline in catalytic potential.
  (7) $$HO^{■}+H_{2}O_{2}\to HO_{2}^{■}+H_{2}O\;$$

In the case of all catalysts (Fe_3_O_4_, GO-Fe_3_O_4_, CoFe_2_O_4_, and GO-CoFe_2_O_4_), there is a gradual increase up to their respective optimum value of oxidant concentration and then a pronounced decrease in the overall efficiency of heterogeneous Fenton’s process. Catalysts (Fe_3_O_4_, GO-Fe_3_O_4_, and GO-CoFe_2_O_4_) showed their highest activity to degrade acetamiprid when oxidant concentration reached up to 14.5 mM. However, in the case of CoFe_2_O_4_, comparatively more oxidant concentration (21.75 mM) was needed, because it has to struggle against the fast recombination of electron-hole pair (e^-^-h^+^ pair), owing to its lower bandgap (than magnetite). At the beginning of the reaction (from 1.16 mM of oxidant to its optimized value), with the increase in H_2_O_2_ concentration, there is a gradual enhancement in the rate of reaction. While the further increase in the oxidant dose decreased the degradation rate due to the scavenging of ^∙^OH radicals by H_2_O_2_ (not needed stoichiometrically) (Equation (8)). Peroxyl radicals formed in the equation do not have enough oxidation potential required for efficient Fenton process, as their rate constant is 2 × 10^4^ M^–1^s^–1^. Therefore, they will lead to a negligible contribution to the degradation process.

(8) $$H_{2}O_{2}+HO^{■}\to HOO^{■}+H_{2}O$$

#### 3.1.3. Effect of Catalyst Dose

The influence of catalyst concentration on its degradation efficiency is represented in Figure 3c. In the present work, variable doses (0–200 mg/L) of catalysts have been studied to achieve optimum degradation. From Figure 3c, it has been observed that a higher value of degradation (for Fe_3_O_4_ 76%, for GO-Fe_3_O_4_ 85%, for CoFe_2_O_4_ 75%, and for GO-CoFe_2_O_4_ 90%) has been achieved at a catalyst dose of 100 mg/L (for each of four catalysts). Initially, a rapidly increasing trend of acetamiprid’s degradation was observed by increasing the photocatalysts up to 100 mg/L, above which it did not promote the degradation potential of catalysts. From 0 to 100 mg/L dose range of catalysts, pesticide removal efficiency increases considerably. As with the increasing concentration of catalyst in the pesticide solution, more active sites will be available for the adsorption (accelerator and target molecule), modifications, and catalytic performance. Enhanced production of hydroxyl radicals and good accommodation of target molecules will be assisted on the grounds of more active sites (Equation (9)). Thus, it can be assumed that at 100 mg/L catalyst dose, there is a perfect balance attained among the three major components of the system (i.e., oxidant concentration, catalyst dose, and pesticide load).

(9) $$HO^{■}+Fe^{2+}\to Fe^{3+}+HO^{-}$$

(10) $$HOO^{■}+Fe^{2+}\to Fe^{3+}+HOO^{-}\;$$

After this, further increase approaching 200 mg/L of catalyst, the degradation rate was decreased because of the catalyst’s agglomeration at its higher concentration. At this point, catalyst sheets start stacking over each other, thus leading to the presence of unexposed active sites, giving no contribution to Fenton’s process. Moreover, this excessive addition of catalysts may lead to the blockage of photon penetration into the solution. In this case, photocatalytic activators have no more appropriate access to light, thus resulting in a negative impact on degradation. Moreover, if the amount of catalyst is enhanced too much and the balance between catalysts to pollutant ratio is disturbed, the in situ produced radicals are scavenged by excess catalytic species.

Graphene oxide-based composites proved more efficient even at a high dosage of catalyst because graphene oxide not only provided conductive support to ferrites but also prevented the catalysts from agglomeration [30].

#### 3.1.4. Effect of Pesticide Load

As ppm levels of pesticide were proved hazardous, a degradation study was also conducted on small amounts (ppm level) of acetamiprid. For this purpose, varying concentrations of acetamiprid (2 to 16 ppm), added in simulated wastewater, were treated using catalysts individually. Results are reported in Figure 3d. Catalysts effectively (80%) degrade 10 ppm solution of pesticide. Nearly 100% of degradation was achieved in the case of 2 to 8 ppm of acetamiprid as reflected by Figure 3d. As the short-lived hydroxyl radicals (lifetime = a few nanoseconds) can only react where they are produced in solution, there will be an enhanced probability of collision between pesticide molecule and the activator when there is an increase in Acetamiprid molecules per unit volume. However, as pesticide dose exceeds beyond 10ppm up to 16ppm, the rate of degradation sharply declines. Because more pollutant molecules are added in wastewater, more active sites are occupied, giving no space for the reaction responsible for the formation of surface-bound reactive oxygen species. Secondly, photons uptake by more concentrated pesticide solution will decrease the availability of photons for activation of catalyst surface (production of hydroxyl radicals over the surface of catalyst). It will also disturb the proper balance in oxidant to pollutant ratio, which imparts a negative impact on degradation reaction. Secondly, the molar extinction coefficient of Acetamiprid is 25 × 10^3^ Lmol^−1^cm^−1^, so increasing Acetamiprid concentration will lead to wastage of incident light for pesticide molecule excitation. Ultimately, the solution will become impermeable to the coming photons.

#### 3.1.5. Effect of Irradiation Time

Up till now, acetamiprid’s degradation reaction conditions, other than time, have been optimized, to get the highest output in terms of degradation. Now, the time, for which pesticide solution is irradiated by UV (254 nm), should be optimized, enhancing the economic feasibility of the process. It is investigated under optimized conditions of pH, oxidant dose, pesticide load, and catalyst dose, by varying irradiation time from 15 to 120 min. At these fixed time intervals, specified in Figure 3e, the aliquots (of treated samples) were taken out and their absorbances reflected the extent of acetamiprid’s degradation. It is clear from Figure 3e that, at the initial stage rate of the degradation process, it sharply increases, but as the process proceeds, the rate becomes slower. Thus, the specific time needed by the active sites to fully degrade the pollutant is 60 min (in the case of all catalysts). The declining rate of reaction reflects that with time during the reaction, (a) hydroxyl radicals are being consumed speedily, while are being regenerated in situ through a slower process; (b) more hydroxyl ions are generated in the medium which affects pH; (c) these generated hydroxyl ions react with ferrous ions and suppress the original Fenton reaction because of limited available active sites on the surface of catalyst; and (d) if ferrous ions are rapidly being oxidized to ferric ions, Fe(OH)_3_ sludge will be produced. This sludge along with other negative impacts will hinder the photon penetration into the solution and their proper access to the catalyst’s surface (where degradation is carried on) [5].

#### 3.1.6. Reusability and Stability of Catalysts

The extraordinary performance of these selected catalysts is also linked to their magnetic property, durability, low catalyst deterioration leading to metal leaching in water bodies, and efficient reusability. The stability of catalysts was investigated by performing successive trials of the used catalysts separately. To do so, used catalysts were magnetically separated by applying an external magnetic field, washed thoroughly with distilled water and ethanol, dried in an oven at 60 °C, weighed, and tested against untreated pesticide solution. The results are shown in Figure 3f. It is evident from the Figure that the catalytic activities of all catalysts (especially GO-based composites) did not drop considerably even after 5 runs. Moreover, the GO-supported catalysts (GO-Fe_3_O_4_, GO-CoFe_2_O_4_) showed enhanced stability and efficiency (more than 65% catalytic power at the 5th run). Thus, the GO-based composite formation enhanced their efficiency, magnetic recoverability, stability, and reusability against acetamiprid remediation. The iron leaching of all catalysts, after each reusability run, was checked using atomic absorption spectroscopy, and the results obtained are presented in Table 1. The low iron leaching values (far below the European Union Standard i.e., 2.0 mg/L [12]) suggest that the heterogeneous catalysts are stable towards metal leaching from solid to liquid state.

- As per EU directives, Fe leaching should not be higher than 2.0 ppm.

#### 3.1.7. Radical Scavenging Test

**Figure 4:**
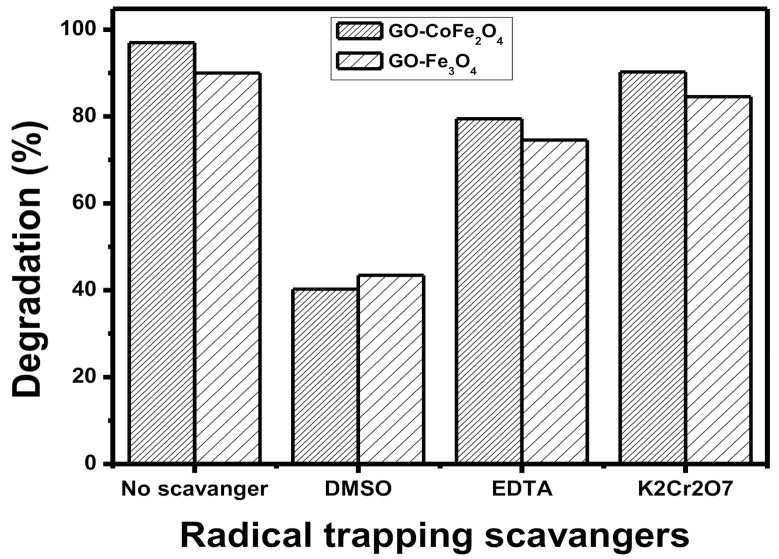
Radical scavenging experiment for the degradation of Acetamiprid under optimized conditions using GO-CoFe_2_O_4_ and GO-Fe_3_O_4_ composites.

The photodegradation of organic pollutants is carried out with reactive species like hydroxyl radicles, holes, electron, etc. Therefore, radical scavenging experiment was performed to find out the key radicals involved in photodegradation of pesticide [31,32,33,34]. The 5 mM of each DMSO (dimethyl sulfoxide), EDTA (ethylene-diamine-tetra-acetate), and K_2_Cr_2_O_7_ (potassium dichromate) were used to scavenge OH radicals, holes, and electron, respectively. The experiments were carried out under UV irradiations. The results obtained are presented in Figure 4. It is obvious from the figure that DMSO is the key radical scavenger in degradation process. The addition of DMSO results decreases in the degradation values from 97 to 40.28 and from 90 to 43.45 for GO-CoFe_2_O_4_ and GO-Fe_3_O_4_, respectively. Similarly, the results showed the little contribution of holes and electrons in photodegradation as the addition of EDTA and K_2_Cr_2_O_7_ represents no considerable reduction in degradation of acetamiprid.

Depending upon the effectiveness of hydroxyl radicals, the possible mechanism for the heterogeneous photo-Fenton reaction is as follows: First of all, pollutants present in wastewater are chemisorbed onto the surface of the catalyst. Hydrogen peroxide reacts with the catalyst’s iron species, and as a result, highly reactive hydroxide radicals are produced (Equations (11) and (12)). These produced hydroxide radicals attack the surface-linked pollutant molecules, resulting in their photocatalytic degradation (Equation (13)).

(11) $$\equiv Fe^{2+}+H_{2}O_{2}\overset{hv}{\to }OH^{■}+OH^{-}+\equiv Fe^{3+}$$

(12) $$\equiv Fe^{3^{+}}+H_{2}O+UV\to \equiv Fe^{2^{+}}+OH^{■}+H^{+}$$

(13) $$OH^{■}+organics_{chemisorbed}\to degraded\;products\;$$

The photo-Fenton-like catalytic process is based on the traditional Fenton process assisted with light radiations. The enhanced acceleration of *OH^■^* ions produced during the Fenton reaction (Equation (11)) can be achieved with the introduction of the light source, which benefits from the synergistic effect of photocatalysis and the Fenton process [35,36].

### 3.2. Kinetics of Photo-Degradation Reaction

Three kinetic models first-order, second-order, and BMG were studied for the degradation of acetamiprid via the Fenton oxidation process (FOP) [37]. These three kinetic models are usually reported for the degradation of organic pollutants owing Fenton oxidation processes (FOP). Gallard and De Laat [38] suggested that FOP should be a simple first order kinetic process, whereas Guedes [39] reported that it should be the second order process. Behnajady and coworkers [40] proposed the mathematical model to predict the chemical reaction kinetics and the performance of FOP under different conditions.

**Figure 5:**
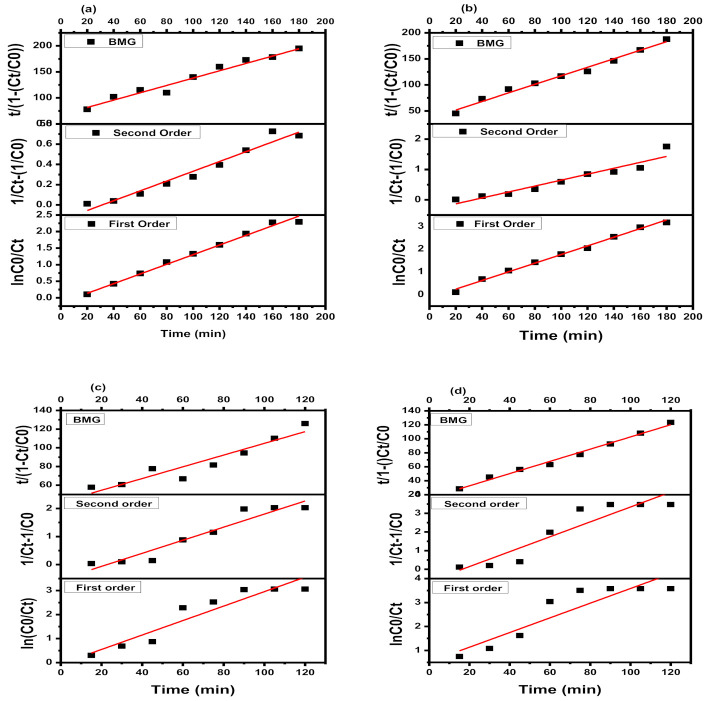
Kinetic model for the degradation of acetamiprid using (**a**) Fe_3_O_4_ and (**b**) GO-Fe_3_O_4_, (**c**) CoFe_2_O_4_ and (**d**) GO-CoFe_2_O_4_.

The expression for each model is presented in Equations (14)–(16). The model graphs are presented in Figure 5. The values of correlation coefficient and rate constants are presented in Table 2.

Considering the correlation coefficient values of the reaction under FOP, the BMG model fitted best to the experimental data as the highest R^2^ was obtained if compared with first-order and second-order models. It can be concluded from Table 2 that the corresponding regression results with a high correlation coefficient (R^2^), from 0.8474 (first order) to 0.9971 (BMG) and 0.8397 (first order) to 0.9911 (BMG) for GO-Fe_3_O_4_ and GO-CoFe_2_O_4_ best suited for BMG kinetic model.

The oxidative degradation of organic pollutants by the Fenton process usually occurs in two steps: the fast and the slower ones. The fast reactive stage was initiated by the availability of hydroxyl radicals, which consumed vigorously during the reaction. In the later stage, ferric ions also react with hydrogen peroxide, and consequently, peroxyl radicals were formed. Peroxyl radicals are other weaker radicals. Apparently, in the reaction hydrogen peroxide is the limiting reactant because with time hydrogen peroxide is consumed rapidly. Here, the rate of formation of hydroxyl radical is slower than its consumption. As the reaction followed two-step patterns, i.e., the very fast and the slower one, neither first-order nor second-order kinetic model could describe the reaction kinetics perfectly. The value of the regression coefficient (R^2^) (0.9971 and 0.9911 GO-Fe_3_O_4_ and GO-CoFe_2_O_4_) justified the fitness of the BMG model.

### 3.3. Optimization through Response Surface Methodology (RSM)

**Figure 6:**
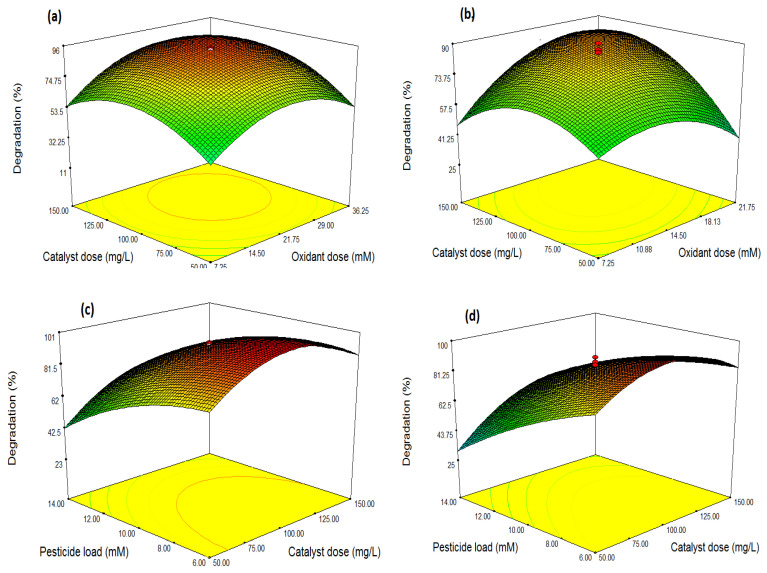

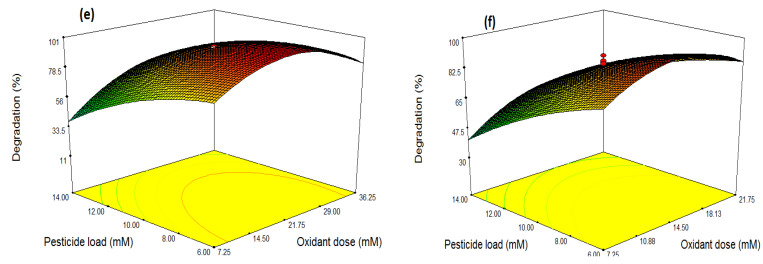
Response surfaces showing the combined effect of (**a,b**) oxidant dose and catalyst dose, (**c,d**) Catalyst dose and pesticide load, (**e,f**) oxidant dose and pesticide load on the degradation of acetamiprid, (**a**,**c**,**e**) using GO-Fe_3_O_4_ and (**b**,**d**,**f**) using GO-CoFe_2_O_4_.

Optimized ranges of influential (selected) parameters were also found out using Response Surface Methodology (RSM). It is a comprehensive statistical tool that helps in identifying the possible interactions among selected parameters. Central Composite Design (CCD) was selected to evaluate the interactive influence of three selected variables (oxidant dose, catalyst dose, and pesticide load) [5]. Ranges of variables were set as predicted by conventional optimization study (single factor influence). CCD model suggested a total of 20 batch experiments (in both cases of composites, i.e., GO-Fe_3_O_4_, GO-CoFe_2_O_4_) to develop the response surfaces. ANOVA (Analysis of Variance) estimated the significance and fitness of the model, variables, and their interactions. For this purpose, the software compares the P-values of each source (variable) and then predicts whether it is significant or not. The summarized results of ANOVA and regression coefficients are shown in Table 3 (in the case of GO-Fe_3_O_4_) and Table 4 (in the case of GO-CoFe_2_O_4_). These represent the individual effects of linear (A, B, and C) and quadratic terms (A^2^, B^2^, C^2^) as well as the first-order interaction effects (AB, AC, and BC). A high correlation between the observed and predicted values is reflected by adjusted R^2^ values (closer to 1) [5]. The influence of parameters individually as well as in combination, on the degradation of the acetamiprid is clear from response surfaces and ANOVA. The adequacy of the model to present the experimental results were justified by significantly large values of R^2^ and adj R^2^. A non-significant lack of fit (*p*-value > 0.05) warranted the reliability of the proposed CCD model. Based on the CCD matrix, different combinations of selected independent variables (catalyst dose (mg/L), oxidant dose (mM), and pesticide load (ppm)) were set to perform a photo-degradation experiment. The response (%age degradation) is reported along with 3D response surfaces (Figure 6). The response (% degradation) varied from 11.6 to 88% (in the case of GO-Fe_3_O_4_) and 13.2 to 90% (in the case of GO-CoFe_2_O_4_). In both cases, the second-order polynomial equation represents the correlation among variables as

Y% _(in case of GO-Fe_3_O_4_)_ = 93.96 + 7.21A + 7.42B − 14.88C + 3.03AB + 6.8AC + 3.2BC − 18.86A^2^ − 17.43B^2^ − 8.24C^2^

Y% _(in case of GO-CoFe_2_O_4_)_ = 84.49 + 5.36A + 8.81B − 17.59C + 12.01AB + 3.19AC + 6.66BC − 13.13A^2^ − 15.92B^2^ − 4.53C^2^

where Y is the response (in terms of % degradation), and A, B, and C represent three selected independent variables, i.e., catalyst dose, oxidant dose, and pesticide concentration, respectively.

#### Optimization and Combined Effects of Independent Variables

The study of contour and response surface plots helped in comprehending the optimization and effects of the selected independent variables on the degradation process. The results are shown in Figure 6a,b. The combined effect of oxidant dose and catalyst dose is shown in Figure 6a. Here, the third variable is kept at an optimized value. It depicts in both cases that there is a threshold value of oxidant to catalyst ratio, above and below which catalysts do not perform efficiently. However, the general trend is that the optimum point (maximum degradation) seemed to be in the mid of the selected ranges. With an increase in catalyst dose, degradation of acetamiprid was increased at moderate oxidant concentration. Very high and very low oxidant doses impart severely low degradation efficiency even if the amount of catalyst is high [5].

The mutual effect of catalyst dose and acetamiprid concentration on the degradation process was studied and reported in Figure 6c,d, keeping oxidant dose fixed. The oxidation potentials of catalysts were enhanced with an increase in catalyst dose and a decrease in acetamiprid concentration. A combined effect in numerical value was also given in terms of the polynomial quadratic, coded equation, as a coefficient. To further enhance the degradation efficiency, more oxidant is required to initiate the oxidative reaction on the surface of the passive catalyst. An interactive study of impacts of oxidant dose and pesticide concentration on degradation of acetamiprid has been represented in Figure 6e,f. It is evident from the Figures that low levels of pesticide concentrations and high levels of oxidant doses support the efficient degradation process.

## 4. Materials and Methods

### 4.1. Materials

Graphite Powder (≥99.99%, Daejung Korea), Sodium Nitrate (≥99.0%, Sigma-Aldrich), Concentrated Sulphuric acid (clear, assay: 99.99%, Sigma-Aldrich), Hydrogen peroxide (30 %, Sigma-Aldrich, Germany), Iron (III) Chloride hexahydrate (99.0%, Daejung Korea) Iron (II) Sulphate heptahydrate (≥98.0%, Sigma-Aldrich), Cobalt chloride hexahydrate (99.0%, Daejung Korea), and Liquid ammonia (Sigma-Aldrich) were used in this study. All chemicals were analytical grade reagents and were used as received without further purification. Distilled water was used throughout the study.

### 4.2. Methods

#### 4.2.1. Preparation of Graphene Oxide and Catalysts

Modified Hummer’s method was used for the synthesis of Graphene oxide, as reported earlier [5]. Here, the graphitic oxide was used as a carbon source, and KMnO_4_, H_2_O_2_, H_2_SO_4_ were used as oxidizing agents. The catalyst was synthesized by ultrasonic impregnation of ferrite particles using Graphene Oxide (GO) as support material where FeCl_3_.6H_2_O and FeSO_4_.7H_2_O act as iron precursors (Fe^3+^ and Fe^2+^, respectively) [41]. One-pot ultrasonically assisted reverse co-precipitation method was followed for the synthesis of magnetite. For this purpose, a fixed amount of ultra-sonicated GO was added into the mixed solution of iron precursors having Fe^2+^/Fe^3+^ mole ratio of 0.5. Under highly alkaline conditions, GO-Fe_3_O_4_ precipitates were prepared. For comparison, Fe_3_O_4_ nanoparticles were also prepared following a similar procedure without addition of GO [42,43]. CoFe_2_O_4_-GO was prepared by adopting one-pot hydrothermal method in the presence of GO suspension [43,44].

#### 4.2.2. Characterization of Catalysts

Catalysts (Fe_3_O_4,_ GO–Fe_3_O_4,_ CoFe_2_O_4,_ GO–CoFe_2_O_4_) were characterized using XRD, FTIR, and SEM analysis. Powder x-ray diffractometer (PW1398, Philips, The Netherlands) with Cu-Kα radiation (λ = 1.5418 Å) was used for XRD analysis. Chemical bonding within the composites was determined by Fourier Transform Infrared spectroscopy (FT-IR, Thermo Nicolet). Scanning Electron Microscope (SEM; JSM5910 JEOL JAPAN) at E = 30 KV was used to investigate microstructures and morphologies of catalysts. The band gap energies of the prepared materials were estimated from the data obtained by UV-Visible spectrophotometer using Tauc plot method.

#### 4.2.3. Degradation Experiment

The photocatalytic experiments were conducted under Ultraviolet light irradiation. UV (254nm) lamps (8 × 18 W) were used as a UV-light source (ZamZam micro technologies ZM144W) for the photo-Fenton degradation of organic compounds at room temperature. The solution samples were placed in an orbital shaker, and the distance between the lamp and test solution was fixed, to maintain constant intensity. Typically, 100 mL of test solution was used in the degradation experiments. The absorbances of pesticide solutions during the photo-Fenton process were measured individually, at regular intervals, using a double beam spectrophotometer. Different parameters like effect of pH (2–8), oxidant concentration (1.16–58 mM), catalyst dose (0–200mg/L), pesticide concentration (2–16 ppm), and contact time (15–120 min), for enhanced degradation of pesticide, were optimized. The range of influencing parameters was selected based on some preliminary experiments and from the literature already reported. The percentage (%) degradation was calculated by using the following formula:

$$Degradation\;\left(\%\right)=\frac{C_{o}-C}{C_{o}}\times 100$$

where *C_o_* and *C* refer to the initial and final concentrations of acetamiprid, respectively.

#### 4.2.4. Kinetic Study of Degradation Reaction

Heterogeneous Fenton-like processes are quite complicated due to the involvement of multiple steps that contribute to the overall degradation reaction [45]. Three kinetic models were applied to the experimental data, i.e., first order, second order, and Behnajady–Modirashahla–Ghanbery (BMG).

Different kinetic models were derived in the form of a linear quadratic equation.

First-order kinetics

(14) $$ln\frac{C_{o}}{C_{t}}=k_{1}.t$$

Second-order kinetics

(15) $$\frac{1}{C_{t}}-\frac{1}{C_{o}}=k_{2}.t$$

Behnajaday–Modrishahla–Ghanbery model

(16) $$t÷\left(1-\frac{C_{t}}{C_{o}}\right)=m+b.t$$

Here, *C_t_*, *C_o_*, *k*_1_, *k*_2_, *t*, *m*, and *b* are the concentration of pesticide at the time “*t*”, at the time “0”; the rate constant for the first-order reaction, for second-order reaction; time and constants in BMG models which are related to the maximum oxidation capacity and kinetics of the reaction, respectively. The fitness of the model was justified through Linear regression analysis (R^2^ value).

#### 4.2.5. Optimization Using Response Surface Methodology (RSM)

Interactions among the influential parameters (classically studied and observed in this paper) were studied through RSM. It is an amalgamated tool involving statistical and mathematical techniques, which empirically evaluates the correlation among several controllable experimental parameters and their reproducible results simultaneously. It is a modeling technique that assists in predicting the simultaneous impact of two or more variables (a) performing statistically designed experiments, (b) estimating the coefficients in a mathematical model, (c) predicting the cumulative response, and (d) checking the adequacy of the model. Central Composite Design (CCD) sufficiently informs about data fitness with a relatively lesser number of runs, so by reducing the overall cost of the experiment [5]. Three independent variables, as oxidant concentration, catalyst dose, and pesticide load, were chosen independent variables, based on the classical study and %age Acetamiprid’s degradation was the dependent response, while other parameters like pH, irradiation time, and UV light were kept constant. There was a total of seventeen experimental runs, consisting of eight factorial points, three central points, and six axial points. Five levels were chosen for each independent variable (i.e., xi = −1.68, −1, 0, 1 and 1.68). Correlation between independent and response variables was interpreted by the Least Square Method (LSM) following the second-order model.

(17) $$Y=\beta_{0}\;+\;\sum_{i=1}^{n}\beta iXi\;+\;+\;\sum_{i=1}^{n}\beta iiXi^{2}\;\;+\sum_{i=1}^{n-1}\sum_{j=i+1}^{n}\beta ijXiXj+\;\varepsilon$$

where Y is the response, *β_0_* is a coefficient with a specific numerical value, *βi*, *βii*, and *βij* belong to the coefficients regarding linear, quadratic, and interaction effects, respectively, while *n* is the number of independent variables and ε (Epsilon) is random error. Statistical computation of F-value, at probability (p) of 0.05, reflects the validity and suitability of polynomial models. Fitness of regression coefficients represented in Analysis of Variance (ANOVA).

## 5. Conclusions

Acetamiprid was selected as a model pollutant. The co-precipitation method has opted for the synthesis of magnetic Fe_3_O_4_ particles, and the hydrothermal method has opted for the synthesis of CoFe_2_O_4._ While respective composite was prepared by doping graphene oxide, prepared by Hummer’s method. Magnetite was proved as a potential catalyst, but composite enhanced its degradation potential. As a result of composite formation, the catalytic activity was enhanced by inhibiting the magnetite’s agglomeration, and thus, active sites are exposed to oxidant as well as pollutants present in water. It also leads to the catalyst’s uniform dispersion. Moreover, the efficient separation was conducted by applying an external magnetic field. This easy post-treatment made it an attractive and feasible technique in terms of large-scale applicability. Thus, the development of such a promising catalyst offers not only high degrading activity to complete mineralization. Low iron leaching, complying with the European Union directives for the discharge of treated waters, contributes to the catalyst’s stability and reusability. Mineralization of 100% can be achieved by focusing on the development of coupled/merged techniques of AOPs, light, and acoustic cavitation because these will help in minimizing the shielding effect, and consequently, light penetration will be enhanced. Thus, wastewater remediation using magnetically assisted separation is an important consideration for cost-effectiveness and less time consumption.

## Figures and Tables

**Table 1:** Stability analysis catalysts in terms of Fe leaching.

Run	Fe_3_O_4_		Fe_3_O_4_-GO		CoFe_2_O_4_		CoFe_2_O_4_-GO	
	Degradation (%)	Iron Leaching (ppm)	Degradation (%)	Iron Leaching (ppm)	Degradation (%)	Iron Leaching (ppm)	Degradation (%)	Iron Leaching (ppm)
**1st**	83	1.28	91	0.62	82.3	1.13	96.43	0.72
**2nd**	73.2	1.22	88.3	0.60	72.3	1.02	89.2	0.68
**3rd**	64.3	1.02	80.3	0.53	70.3	0.94	79.3	0.62
**4th**	59.6	0.94	75	0.50	54.2	0.88	70.2	0.57
**5th**	55	0.79	72	0.44	50.1	0.82	67.6	0.46

**Table 2:** Values of the coefficient of correlation and rate constant of acetamiprid degradation using different catalysts.

Catalysts	First-Order		Second-Order		BMG		
	R^2^	K_1_(min^−1^)	R^2^	K_2_(L µmol^−1^min^−1^)	R^2^	m	b
Fe_3_O_4_	0.8971	0.0178	0.9891	0.0096	0.9767	24.396	0.8653
GO-Fe_3_O_4_	0.8474	0.0193	0.9679	0.0193	0.9971	9.7356	0.9546
CoFe_2_O_4_	0.8946	0.0233	0.9207	0.0233	0.9008	41.982	0.6278
GO-CoFe_2_O_4_	0.8397	0.0306	0.8688	0.0400	0.9911	15.267	0.8745

**Table 3:** ANOVA results of the quadratic model for degradation of acetamiprid using GO-Fe_3_O_4_.

Source	Sum of Square	df	Mean Square	F Value	*p*-Value (Prob>F)	
Model	1572.94	9	1747.22	2527.16	<0.0001	**Significant**
A-Oxidant Dose	327.41	1	327.41	473.56	<0.0001	
B-Catalyst Dose	1492.72	1	1492.72	2159.05	<0.0001	
C-Pesticide load	2508.02	1	2508.02	3627.57	<0.0001	
AB	632.72	1	632.72	915.16	<0.0001	
AC	1211.65	1	1211.65	1752.52	<0.0001	
BC	388.01	1	388.01	561.21	<0.0001	
A2	7396.43	1	7396.43	10698.12	<0.0001	
B2	2418.69	1	2418.69	3498.36	<0.0001	
C2	489.09	1	489.09	707.41	<0.0001	
Residual	6.91	10	.069			
Lack of Fit	4.95	5	.99	2.51	0.1674	**Non-significant**
Pure Error	1.97	5	.39			
Cor Total	15,731.86	19				
Std. Dev.		0.83	R-squared		0.99996	
Mean		65.48	Adj. R-squared		0.9992	
C.V.%		1.27	Pred. R-squared		0.9971	
PRESS		45.46	Adeq. precision		148.021	

**Table 4:** ANOVA results of quadratic model for degradation of acetamiprid using GO-CoFe_2_O_4_.

Source	Sum of Square	df	Mean Square	F Value	*p*-Value (Prob>F)	
Model	12,924.55	9	1436.06	20.37	<0.0001	**Significant**
A-Oxidant Dose	392.60	1	392.60	5.57	0.0399	
B-Catalyst Dose	1060.05	1	1060.05	15.04	0.0031	
C-Pesticide load	4225.51	1	4225.51	59.95	<0.0001	
AB	1164.76	1	1164.76	16.52	0.0023	
AC	81.47	1	81.47	1.16	0.3076	
BC	354.71	1	354.71	5.03	0.0487	
A2	2482.81	1	2482.81	35.22	0.0001	
B2	3651.90	1	3651.90	51.81	<0.0001	
C2	296.29	1	296.29	4.20	0.0675	
Residual	704.86	10	70.49			
Lack of Fit	497.67	5	99.53	2.40	0.1791	**Non-significant**
Pure Error	207.19	5	4144			
Cor Total	13,629.40	19				
Std. Dev.		8.40		R-squared	0.9483	
Mean		61.56		Adj. R-squared	0.9017	
C.V.%		13.64		Pred. R-squared	0.6815	
PRESS		4340.44		Adeq. precision	14.640	
